# Dairy heifers have an earlier increase in serum pregnancy-specific protein B compared with lactating dairy cows. Is this an indicator of earlier conceptus attachment?

**DOI:** 10.3168/jdsc.2021-0198

**Published:** 2022-05-21

**Authors:** E.L. Middleton, T. Minela, M. Ahearne, H. Arnold, A. Santos, J.R. Pursley

**Affiliations:** Department of Animal Science, Michigan State University, East Lansing 48824

## Abstract

This study was designed to use within-individual daily increases in circulating pregnancy-specific protein B (PSPB) to determine time to increase in serum PSPB in nulliparous heifers and multiparous dairy cows following artificial insemination (AI). Weekly cohorts of lactating Holstein cows (n = 56) averaging 122 ± 7 d in milk at AI and nulliparous Holstein heifers (n = 23) averaging 16 ± 0.24 mo old at AI were used in this study. Lactating cows received AI to either Ovsynch (n = 38) or at ~12 h following observed standing estrus (n = 18). All heifers were inseminated ~12 h after observed standing estrus. Blood samples for measurement of PSPB were collected daily from d 15 through d 35 postovulation. Nulliparous heifers had increases in serum PSPB earlier compared with both primiparous and multiparous cows. Day of increase in serum PSPB was defined as the day serum PSPB optical density levels initially increased ≥10% from baseline and continued to increase from baseline of ≥10% the following 2 d. Average PSPB were greater in pregnant heifers compared pregnant cows from d 23 through 29. Early lactation nonpregnant cows maintained greater average optical density of serum PSPB from 15 to 35 d postovulation compared with nonpregnant heifers (n = 38). In summary, fertility differences in heifers versus lactating cows may be due to the differences in timing of increases in serum PSPB. This appears to be one of the first publications that used daily PSPB sampling to investigate possible differences in fertility in heifers versus lactating cows.

## Highlights

• Increases in PSPB began on day 20 postovulation in nulliparous Holstein heifers.
• Nulliparous heifers had earlier increases in PSPB compared with lactating cows.
• Initial daily increases in PSPB may be a robust marker for conceptus attachment.

## Graphical Abstract

**Figure:**
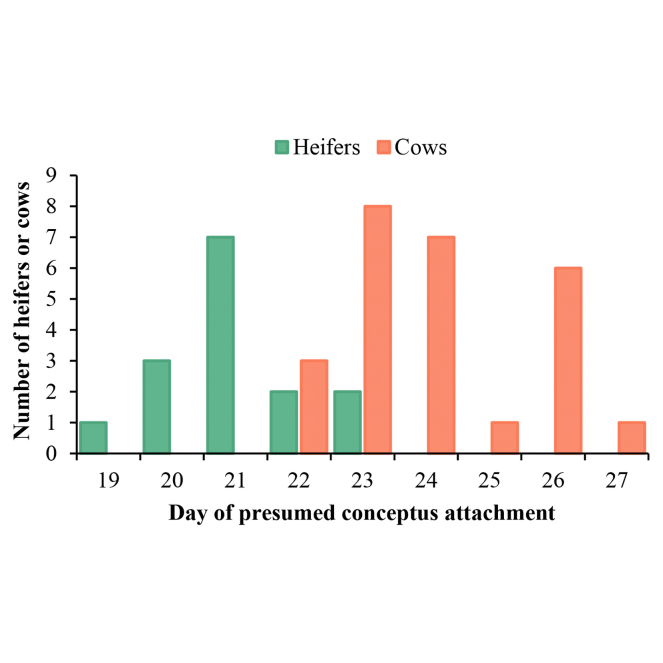
**Summary:** Lactating dairy cows differ from primiparous heifers in a number of different fertility parameters. Differences in timing of increases in serum pregnancy-specific protein B (PSPB) between cows and heifers may help to gain an understanding of some of these parameters. Nulliparous dairy heifers underwent increases in serum PSPB earlier compared to lactating Holstein dairy cows. Day 28 PSPB levels were greater and less variable in pregnant heifers versus lactating cows that had increases in serum PSPB >23 days postovulation. The figure shows the distribution of numbers of nulliparous Holstein heifers and primiparous and multiparous Holstein lactating cows (n = 41) on day of increase in serum PSPB. Day of increase in serum PSPB was considered day of attachment and was defined as day of at least a ≥10% increase in PSPB optical density levels for three consecutive days from a baseline preattachment average of day 15, 16, and 17 post-ovulation.

Conceptus attachment in cattle has been estimated to begin between d 18 to 22 post-AI of development in both morphological (Wathes and Wooding, 1980; Guillomot and Guay, 1982) and genomic studies (Yamakoshi et al., 2012). But there is a paucity of information regarding the precise timing of conceptus attachment in cattle and, in particular, how it relates to fertility in dairy cattle.

The disparity in fertility between nulliparous heifers and multiparous dairy cows is a well-documented phenomenon (Wiltbank et al., 2011). Heifers appear to have an advantage in embryonic development compared with cows. Embryo quality was greater in heifers compared with lactating cows on d 5 after ovulation (Sartori et al., 2002). Green et al. (2010) found differences in interferon stimulating gene expression in younger versus older cows and hypothesized younger cows may have larger conceptus sizes. The bovine conceptus increases in size over 1,000-fold between the hatched blastocyst and filamentous embryo stages. The increase in conceptus size is mostly due to expansion of trophectoderm and endoderm (Spencer et al., 2016). This expansion of embryonic external membranes is required for adhesion, attachment, and consequently maternal recognition of pregnancy (Blomberg et al., 2008). Berg et al. (2010) found greater conceptus size in nulliparous heifers when compared with multiparous cows. Thus, heifers appear to have a distinct advantage in embryonic development leading up to maternal recognition compared with cows (Berg et al., 2010).

Binucleate cells release several glycoproteins that are associated with pregnancy (**PAG**) into the maternal circulation, such as pregnancy-specific protein B (**PSPB**; Zoli et al., 1992a; Kiracofe et al., 1993; Roberts et al., 1996). Levels of PAG are higher in maternal than in fetal circulation (Zoli et al., 1992a). Thus, maternal circulating levels of PSPB could be used as a direct indicator of fusion of binucleate cells with uterine epithelial cells, and as a potential marker for conceptus attachment. Assays for PAG in maternal circulation, including the measurement of PSPB, are available as determinants of pregnancy in cattle (Humblot et al., 1988). Pregnancy cannot be accurately diagnosed in lactating cows with a single PSPB sample until 28 d post-AI (Zoli et al., 1992b; Humblot, 2001; Piechotta et al., 2011) because of variability between cows (Sasser et al., 1986). Nulliparous heifers, however, can accurately be diagnosed for pregnancy with a single PSPB sample taken 25 d post-AI (Humblot et al., 1988). Recent data indicated that the use of serum levels of PSPB within individual cows before and after estimated time of increase in serum PSPB (Martins et al., 2018; Middleton and Pursley, 2019) is a robust method to detect nonpregnant cows. It was determined that a 10% or greater increase from a preattachment baseline was accurate in diagnosing pregnancy at 24 d post-AI (Middleton and Pursley, 2019). Using this method, in addition to collecting daily samples of PSPB, may help to distinguish differences in fertility in heifers and cows and possibly provide an estimate for time to conceptus attachment in cattle.

The objective of this study was to determine the day of increase in PSPB levels in nulliparous dairy heifers versus lactating dairy cows utilizing daily measurements before and after expected time of conceptus attachment.

This study was conducted at Michigan State University's Dairy Cattle Teaching and Research Center (East Lansing). Lactating cows were housed in either a covered freestall or tiestall barn and milked twice daily. Heifers were housed in a covered freestall barn. Both lactating cows and heifers were fed a TMR consisting of corn, soybean meal, alfalfa, and corn silage formulated to meet nutrient recommendations (NRC, 2001) with free access to water and minerals. The Institutional Animal Care and Use Committee at Michigan State University approved all animal handling and procedures.

Weekly cohorts of lactating Holstein cows (n = 56) averaging 122 ± 7 DIM at AI and nulliparous Holstein heifers (n = 23) averaging 16 ± 0.24 mo old at AI were used in this study. The number of pregnant heifers/cows per group needed at 0.85 power to determine a 2-d difference in day of increase in PSPB was n = 15. Lactating cows received AI to either Ovsynch (n = 38; Pursley et al., 1995) or at ~12 h following observed standing estrus (n = 18). All heifers were inseminated ~12 h after observed standing estrus. Estrus detection was performed visually in the early morning and late afternoon. Blood samples for measurement of PSPB were collected daily from d 15 through d 35 postovulation. Reference d 0 for PSPB was the estimated day of ovulation (**OV**). Day of ovulation was estimated to be 24 h after either final GnRH of Ovsynch or initial observation of standing estrus (Pursley et al., 1995; Walker et al., 1996).

Blood samples were collected from the coccygeal vein or artery by trained laboratory personnel using chilled heparinized tubes. The serum portion was separated immediately after collection by centrifugation at 1,600 × *g* for 20 min at 4°C and stored at −20°C for later analyses. Samples for PSPB were analyzed in duplicate by an independent laboratory using the BioPRYN ELISA assay (BioTracking LLC). Each set of individual heifer or cow daily samples collected from 15 to 35 d postovulation was analyzed on an individual plate. Intra- and interassay coefficients of variation were 5.4 and 10.3%. Levels of PSPB were expressed in units of optical density (**OD**), which was the typical way to express outcomes using the BioPRYN assay. We chose the BioPRYN assay because it is a common assay to test for pregnancy beginning around d 28 post-AI utilizing a single blood sample and is widely used throughout the United States.

Serum PSPB OD from d 15, 16, and 17 post-OV were averaged for each cow and heifer to obtain a baseline PSPB value. Sensitivities and specificities of day of attachment were calculated for increases of 1, 2.5, 5, 7.5, 10, 12.5, 15, and 20% from baseline PSPB for 3 consecutive days. A single day increase had lower specificity compared with 2 or 3 consecutive days. The most conservative estimate of increases from baseline for 3 consecutive days with 100% positive predictive value was chosen as the cut-off. Day of increase in serum PSPB was defined as the day serum PSPB OD levels initially increased ≥10% from baseline (Middleton and Pursley, 2019) and continued to increase from baseline of ≥10% the following 2 d. Cows and heifers were categorized into “early” or “late” increases in serum PSPB if increases occurred before or after d 23 post-OV, respectively.

Cows and heifers were diagnosed for pregnancy 36 d post-OV using transrectal ultrasound with a 7.5-MHz linear transducer (Aloka 500 SSD; Corometrics Medical Systems Inc.). Pregnancy was diagnosed by visualization of an embryo with a heartbeat.

All information was recorded in an Excel (Microsoft Corp.) spreadsheet for organization before statistical analysis. The FREQ procedure was used to estimate sensitivity, specificity, and negative and positive predictive values of various thresholds of percentage increase of PSPB as a marker of increases in serum PSPB. Each threshold's outcome (increase or no increases in serum PSPB) was compared with the reference pregnancy diagnosis (pregnant or nonpregnant) at d 36 post-OV. Days to increases in serum PSPB was analyzed with linear mixed models implemented by the MIXED procedure of SAS (version 9.4, SAS Institute Inc.). The models either included parity (nulliparous, primiparous, and multiparous), stage of PSPB increase (heifer-early, cow-early, and cow-late) or AI-type (Ovsynch or estrus). Levene's test was used in PROC GLM to test for equality of variances between heifers and cows in days to increases in serum PSPB. Differences in PSPB concentration at d 28 post-OV between stages of when PSPB increased were determined with PROC MIXED. The model included stage of PSPB increase as the fixed effect. The LSMEANS statement was used to slice any significant fixed effects. Daily PSPB concentrations were analyzed using the MIXED procedure of SAS with the REPEATED statement. The model included the fixed effects of time (d 15 to 35) and cow/heifer (pregnant and nonpregnant), and their interaction. Cow/heifer was also specified in the SUBJECT option. A first-order autoregressive covariance structure was used. Nonpregnant cows were divided into tertiles of DIM at day of ovulation to determine differences in PSPB compared with nulliparous nonpregnant heifers. The model was fitted with PROC MIXED and included DIM tertiles as the fixed effect and averages of either the first or last 5 d of sampling as the response variable. Relationships between 2 continuous variables were analyzed using the CORR procedure of SAS and tested by the Pearson correlation test. The MEANS procedure of SAS was used to report means ± standard errors of continuous variables.

Twenty-six of 56 lactating cows and 15 of 23 nulliparous heifers became pregnant in this study. Figure 1 describes serum levels of PSPB from d 15 to 35 post-OV for both pregnant and nonpregnant cows and heifers. No heifers and only n = 1 cow lost pregnancies before the d-36 pregnancy diagnosis. There was no difference in average ± SEM PSPB OD in the 3-d baseline period (d 15, 16, and 17) for calculating day of increase in serum PSPB in pregnant versus nonpregnant cows (*P* = 0.86) or pregnant versus nonpregnant heifers (*P* = 0.99). Circulating levels of PSPB in heifers increased earlier and remained greater compared with lactating cows until d 29 postovulation (Figure 1). So, in comparison, daily changes in PSPB may be a reasonable marker for timing of attachment. Average PSPB was greater in pregnant heifers versus cows from d 23 through 29. In agreement, previous data indicated that pregnant heifers had detectable levels of PSPB earlier than pregnant cows (Humblot et al., 1988; Filho et al., 2020). It is plausible that differences in time to when PSPB increases in heifers versus cows may differ based on differences in fertility, pregnancy loss, and differences in progesterone (Sartori et al., 2002). In this regard, the greater progesterone environment in heifers may affect the uterine environment and its impact on elongation of the embryo.

**Figure 1:**
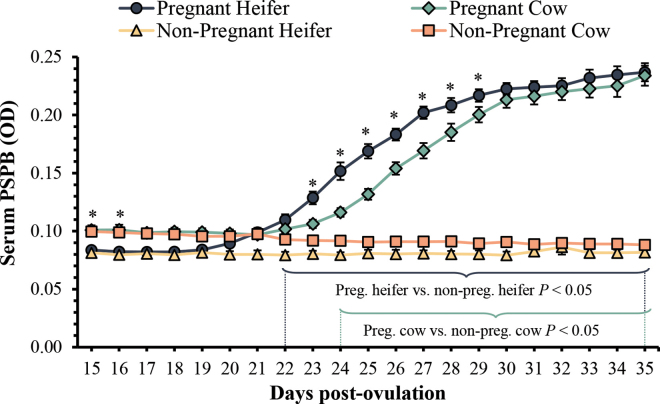
Differences in average ± SEM daily pregnancy-specific protein B (PSPB) optical density measurements from d 15 to 35 postovulation for both pregnant and nonpregnant lactating Holstein cows (n = 56) and nulliparous Holstein heifers (n = 23). The blue bracket signals significant differences in serum PSPB when comparing pregnant heifers with nonpregnant heifers (*P* < 0.05 between d 22 and 35; Tukey-Kramer). The green bracket signals significant differences in serum PSPB when comparing pregnant cows with nonpregnant cows (*P* < 0.05 between d 24 and 35; Tukey-Kramer). The symbol * denotes significant differences in serum PSPB when comparing pregnant heifers with pregnant cows.

The key outcome in this study describes a stark difference in time to increase in serum PSPB in heifers versus lactating dairy cows. Nulliparous heifers underwent increases in serum PSPB earlier compared with both primiparous and multiparous cows (Figure 2). Average day to increase in serum PSPB in heifers coincided closely to timing of attachment described in previous studies (Wathes and Wooding, 1980; Guillomot and Guay, 1982; Yamakoshi et al., 2012). This difference in time to increase in serum PSPB may be directly related to differences in fertility between cows and heifers. Greater fertility observed in heifers compared with lactating dairy cows has been contributed to greater circulating concentrations of progesterone (Sartori et al., 2002, 2004; Wiltbank et al., 2006). Circulating concentrations of progesterone may play an important role in conceptus elongation and endometrial regulation (Carter et al., 2008; Clemente et al., 2009). Uncompromised elongation may be an explanation for differences in time to attachment between heifers and cows. Berg et al. (2010) reported that embryos retrieved from heifers had an advantage in length compared with cows in the early elongation period, and Green et al. (2010) reported greater interferon-tau stimulated gene expression in pregnant heifers compared with pregnant cows on d 18 post-AI. These findings may indicate that proper development of the elongating embryo may determine time to attachment.

**Figure 2:**
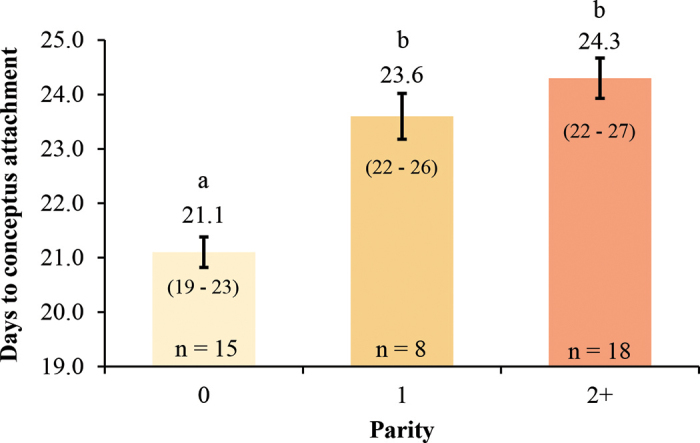
Differences in average ± SEM days to increase in serum pregnancy-specific protein B (PSPB), defined as days of at least a ≥10% increase in PSPB optical density levels from a baseline preattachment average of d 15, 16, and 17 postovulation, between nulliparous Holstein heifers and primiparous and multiparous Holstein lactating cows. Values inside the bars and in parentheses represent the range of days to increase in serum PSPB within each parity. Bars with different letters (a,b) differ significantly (*P* < 0.01).

Variation in serum concentrations of PSPB on d 28 post-OV was less in heifers compared with cows with early and late increases in serum PSPB (Figure 3). Average serum levels of PSPB on d 28 postovulation in pregnant heifers and cows reflect the difference in time to attachment. Heifers had greater (*P* = 0.04) levels of circulating PSPB on d 28 post-OV compared with cows with late increases in serum PSPB.

**Figure 3:**
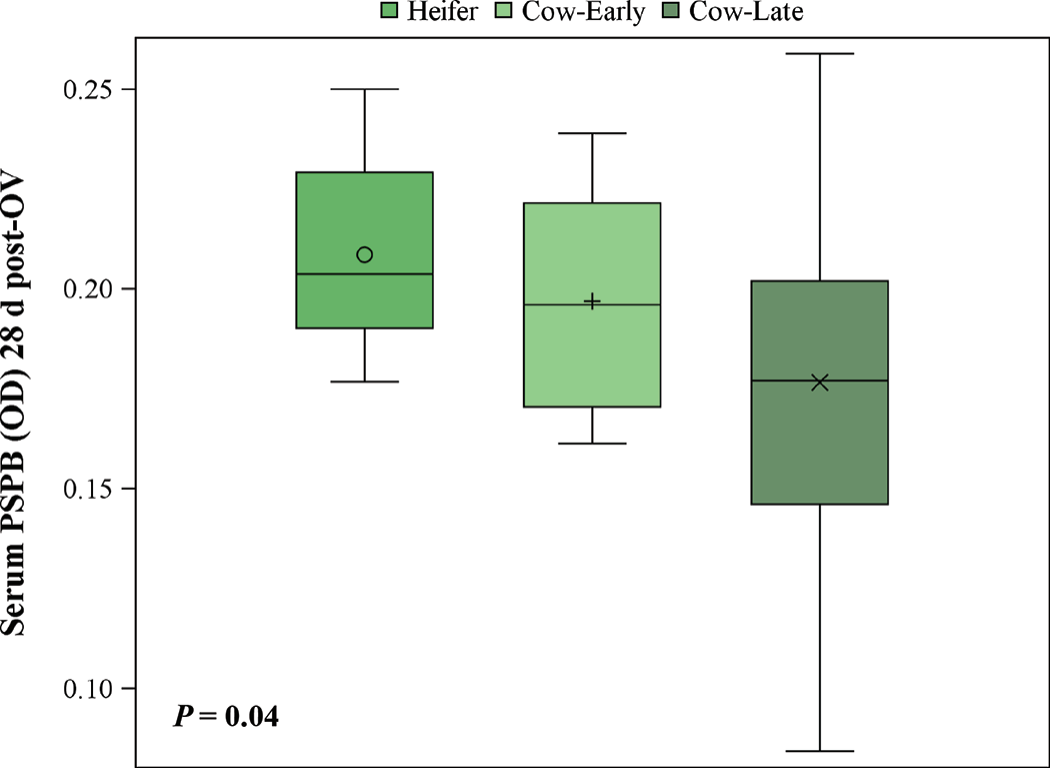
Description of the variation in levels of pregnancy-specific protein B (PSPB; optical density, OD) displayed in boxplots of serum PSPB (OD) measured on d 28 postovulation (post-OV) for pregnant nulliparous heifers (n = 15), pregnant lactating cows that underwent early increases in serum PSPB from d 20 to 23 post-OV (Cow-Early; n = 11), and pregnant lactating cows that underwent late increases in serum PSPB from 24 to 27 post-OV (Cow-Late; n = 15). Day of increase in serum PSPB was defined as the day serum PSPB OD levels initially increased ≥10% from baseline and continued to increase from baseline of ≥10% the following 2 d. The box encompasses the interquartile range (25th through 75th percentile) with the vertical line representing the median value. The whiskers indicate the minimum and maximum values. The symbols within the boxes represent the mean. The *P*-value refers to the fixed effect of stage of PSPB increase classification.

There was no relationship (*P* = 0.31) between day of increase in serum PSPB and insemination type (Ovsynch vs. standing estrus) in lactating cows. Of n = 17 pregnant cows timed-inseminated using Ovsynch, n = 6 underwent early increases in serum PSPB and n = 11 underwent late increases in serum PSPB. Of the n = 9 pregnant cows inseminated to a standing estrus n = 5 underwent early increases in serum PSPB and n = 4 underwent late increases in serum PSPB. There was no relationship between time to increase in serum PSPB and DIM at ovulation in lactating cows (*P* = 0.11; n = 26).

Differences in serum concentrations of PSPB in nonpregnant cows and nulliparous heifers were dependent on DIM of the nonpregnant cows on day of ovulation. Cows that were early in lactation (first tertile; 55 to 66 DIM) had greater PSPB compared with nulliparous heifers during the first (*P* < 0.01) and last (*P* = 0.02) 5 d of sampling. Serum PSPB in cows that were later in lactation (tertiles 2 and 3; 73 to 134 and 153 to 231 DIM, respectively) did not differ from nulliparous heifers during the first 5 d of sampling (tertile 2 vs. heifers *P* = 0.19; and tertile 3 vs. heifers *P* = 0.28) or the last 5 d of sampling (tertile 2 vs. heifers *P* = 0.47; and tertile 3 vs. heifers *P* = 0.47).

Serum concentrations of PSPB on d 28 post-OV were considerably more variable in cows that underwent late increases in serum PSPB (≥24 d post-AI) than cows that underwent early increases in serum PSPB (<24 d post-OV) or heifers (Figure 3). All cows that underwent early increases in serum PSPB and heifers would have been diagnosed either as pregnant or a high recheck with the commercially available BioPRYN assay. Cows that underwent late increases in serum PSPB, however, had greater risk of being diagnosed as a low recheck or open. Low levels of PSPB at varying time points have been reported to be indicative of future pregnancy loss (Gábor et al., 2016; Martins et al., 2018; Middleton and Pursley, 2019). Variation between lactating cows at d 28 is likely due to variation in time to attachment.

Is it possible that the rise in PSPB described in this paper is a direct marker of conceptus attachment? Localization of modern PAG (such as PSPB) is exclusively associated with cellular components during the process of trophoblast-endometrium adhesion (Wooding et al., 2005) and mostly located in the cotyledonary region (Touzard et al., 2013). Wooding et al. (2005) identified PAG on the outer surface or within vesicles of apical trophoblastic microvilli cells, which are in close proximity with the uterine epithelial microvillar surface. In addition, these authors reported that these PAG are not at all expressed in areas of less definitive contact, such as interplacentomal regions. Green et al. (1998) also argued that the presence of PAG in the maternal circulation is a consequence of their release close to maternal capillaries and the invasive nature of the binucleate cell. Overall, these findings underline the localization and potential form of transport of these proteins into the maternal circulation. Based on published literature, it seems plausible that the early increase in PSPB herein reported was a result of the early events associated with conceptus attachment. To our knowledge, the occurrence of another physiological event during early pregnancy that could yield such an increase in PSPB in maternal circulation is unlikely.

In summary, nulliparous dairy heifers underwent increases in serum PSPB earlier than primiparous and multiparous lactating dairy cows. Variation in PSPB between lactating dairy cows on d 28 post-OV, the earliest time that a single sample can be used to predict pregnancy, can likely be explained by timing of conceptus attachment in this study. This appears to be the first published study reporting differences in timing of increase in serum PSPB in dairy heifers compared with less fertile lactating dairy cows.
